# Nanoparticles Induced Biomimetic Remineralization of Acid-Etched Dentin

**DOI:** 10.30476/dentjods.2024.98928.2117

**Published:** 2024-12-01

**Authors:** Devalla Venu Babu, Srinidhi V. Ballullaya, Pushpa S, Neha Taufin, Pilli Sai Naveen

**Affiliations:** 1 Dept. Conservative Dentistry and Endodontics, St.Joseph Dental College, Duggirala, Eluru, Andra Pradesh, India

**Keywords:** Biomimetics, Biomineralization, Chitosan, Calcium phosphate, Dentin, Hydroxy apatite, Nanoparticles

## Abstract

**Statement of the Problem:**

Dentin bonding with etch-and-rinse adhesives involves demineralizing the 5-8µm of the surface dentin to create micro space for resin infiltration. The presence of continuous fluid movement in dentin tubules and positive pulpal pressure prevents complete water replacement by resin monomers. This results in areas of demineralized dentin, which contain collagen fibers without resin infiltration. The exposed collage fibers are subjected to enzymatic degradation leading to less durable hybrid layer.

**Purpose:**

The aim of this study was to evaluate the remineralizing effect of the nanoparticles on the resin dentin bonding interface.

**Materials and Method:**

The three experimental remineralizing nanoparticles were characterized for their morphology, size, and composition. A total of 48 extracted non-carious human third molar teeth were sectioned at 2 mm below the cemento enamel junction. Class I cavity was prepared and the tooth samples were placed in an intra pulpal pressure simulation device. After etching of the prepared cavity, the samples were randomly divided into four groups (n=10) as follows: (1) control group(c) (n=10) (2) Nano-hydroxyapatite (nHAP) (n=10) (3) Chitosan-nanohydroxyapatite (Chi-nHAP) (n=10) (4) Mesoporous silica-hydrox-yapatite (MS-nHAP) (n=10). After 30 days remineralization period, the samples were evaluated for micro tensile bond strength, hybrid layer morphology, and mineral composition of the hybrid layer. The results were analyzed statistically by one-way ANOVA and Tukey's multiple post hoc tests.

**Results:**

Scanning electron microscopic observation of nanoparticles revealed irregular particle shapes with calcium phosphate ratio of 1.60. The zeta analyzer showed a mean diameter of 161.0 nm, 323.0nm, 185.0nm for nHAP, Chi-nHAP, and MS-nHAP respectively. Post hoc Bonferroni test revealed significantly higher bond strength for nHAP, Chi-nHAP, and MS-nHAP when compared to control group. MS-nHAP resulted in the uniform deposition of apatite crystal on the surface without any evidence of dentinal tubules openings and had higher mineral to matrix ratio compared to other groups.

**Conclusion:**

MS-nHAP nanoparticles can be considered as a reliable source of calcium and phosphate for biomimetic remineralization of hybrid layer. Application of nanoparticle remineralization precursors before application of dentin bonding agents results in remeralization of exposed collagen fibers thereby improving the clinical longevity of hybrid layer.

## Introduction

The hybrid layer degradation has been an important reason for failure of composite resin restoration, which has been addressed by use of matrix metalloproteinase inhibitors, collagen cross-linkers, changes in monomer chemistry and biomimetic remineralization [ 1 ]. 

Biomimetic remineralization of dentine cannot be achieved by classical ion-based remineralization because of the absence of seed crystals and presence of significant amount of organic matrix as compared to enamel [ 1 ]. Tay *et al*. [ 2 - 3 ] reported the guided tissue remineralization of partially demineralized dentine (acid-etched dentin). They demonstrated intrafibrillar and interfibrillar remineralization of the collagen matrix by employing a non-classical particle mediated crystallization pathway.

The use of nanoparticles has opened up a new avenue for remineralization of acid affected enamel and dentin, which provides a means of replacing lost minerals or as a carrier of ions (Ca,P,F) that are released following particle dissolution. A significant challenge for the use of nanomaterials for remineralization is to achieve an effective and deep infiltration into demineralized collagen without precipitation on the surface. The studied nanomaterials are nano-sized calcium fluoride, nanoparticulate hydroxyapatite, nano-sized carbonated apatite, carbonated hydroxyapatite nanocrystals, and nanoparticulate bioactive glass [ 4 ].

Mineralization of collagen fibrils using solution-based systems containing biomimetic analogs of matrix proteins to stabilize supersaturated calcium phosphate solutions have been predictably achieved *in vitro*. Solution-based systems have limitations when used for in-situ remineralization of human hypo mineralized tissues because the periodic replenishment of the mineralizing solution is infeasible [ 5 ]. A carrier-based platform designed for delivering mineral precursors would be highly desirable. The solution-based biomineralization concept may be translated via the use of mesoporous silica nanoparticles into a carrier-based delivery system to release polymer-stabilized amorphous calcium phosphate (ACP) precursors for the biomineralization of collagen fibrils [ 5 ].

A new therapeutic precursor solution was formulated containing nanoparticles of hydroxyapatite, non-collagenous protein analogs and a phosphate bonded template analog with or without carrier-based. The null hypothesis was that there would be no difference in the micro tensile bond strength for the solution-based and carrier-based remineralization delivery system compared to control group.

## Materials and Method

Forty-eight intact mandibular first molars extracted from patients of age 30-45 yrs for periodontal reasons were collected. All the teeth were stored in 0.5% thymol for not more than one month. This study was approved by the Institutional Review Board (ref no:1717/EA2b/ MDS/2017). 

### Synthesis of nanoparticles and preparation of the experimental remineralization solutions

Nano hydroxyapatite (nHAP), Chitosan with nano-hydroxyapatite (Chi-nHAP)and Mesoporous silica with nano-hydroxyapatite (MS-nHAP)were synthesized at the Nano Research Lab, Haryana, India, as per the method of Chandrashekar *et al*. [ 8 ] Calvo *et al*. [ 9 ] Xuetao *et al*. [ 10 ] and the composition of the three experimental formulations are presented in Table 1.

**Table 1:** Composition of experimental nanoparticles solution for biomimetic remineralization

Solution-based remineralization (nHAP)	Functional group remineralization solution (Chi-nHAP)	Carrier-based remineralization solution (MS-nHAP)
Nano hydroxyapatite 10 wt%	Phosphorylated Chitosan	Mesoporous silica 30wt%
	10 wt% nano-hydroxyapatite	10 wt% nano-hydroxyapatite
500 µg polyacrylic acid	500 µg polyacrylic acid	500 µg polyacrylic acid
200µg polyvinyl phosphonic acid	200µg polyvinyl phosphonic acid	200µg polyvinyl phosphonic acid
Ethanol as vehicle	Ethanol as vehicle	Ethanol as vehicle

Hydroxyl apatite nanoparticles (nHAP); chitosan + hydroxy apatite nanoparticles (chi-nHAP); mesoporous silica+ hydroxy apatite nanoparticles (MS-nHAP)

### Characterization of the experimental formulations

The three experimental remineralization formulations were characterized for size, charge, and molecular weight using a Zeta analyzer. The functional groups and composition were confirmed by Fourier-transform infrared spectroscopy (FTIR). Scanning electron microscopic (SEM-EDX) examination was carried out to know the morphology of nanoparticles and the elemental composition at 2500×.

### Specimen preparation

Forty-eight permanent mandibular molars teeth were selected based on their mesiodistal and buccolingual dimensions and the roots of all the teeth were removed by sectioning at 2mm below the cementoenamel junction using slow speed water-cooled diamond saw. Class I cavity was prepared with a high-speed handpiece with water coolant. Regarding the dimensions of the tooth preparation, the width was one-half of the inter-cuspal distance and the depth extended 3mm below the central pit. Care was taken to maintain the remaining dentin thickness at 1.89±0.23mm [ 6 ], measured with a digital micrometer. After preparing class I cavity preparation, the pulp chamber was placed in an ultrasonic cleaner containing 5.25% NaOCl (Vishal dento care pvt.Ltd, Gujarat, India) and 17% ethylene diamine tetra acetic acid EDTA (Desmear, Anabond Stedman Pharma Research pvt Ltd, Tamilnadu, India.) alternatively for 10 mins to open up the dentinal tubule. Lastly, the teeth were rinsed with saline to remove traces of EDTA.

### Description of the Intra pulpal pressure simulation (IPPS) model

The model consisted of chamber with polyvinyl chloride tubing, submersible motor, circular disc for specimen attachment and pressure gauge. Polyvinyl chloride (PVC) tubing of diameter 4 mm was used for passage of simulated body fluid. T-shape joint was attached to the 4 mm horizontal PVC tube over which a circular disc of 6 mm diameter was secured using iron nipples of 3mm diameter. A tooth was attached to the disc using cyanoacrylate glue.

**Figure 1:**
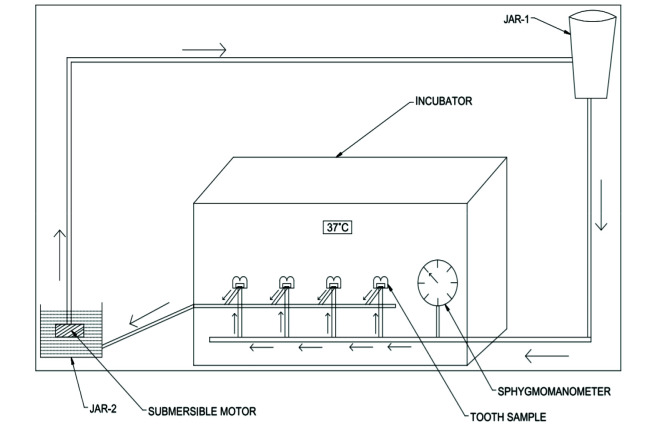
Intra pulpal pressure simulation model with tooth mounted on circular disc and temperature maintained at 37ºc

On the buccal surface of each specimen, a hole was drilled in the middle of the pulp chamber corresponding to 18-gauge needle with a bur. The 18-gauge needle was passed to the tooth through the hole and sealed using cyanoacrylate glue. The needle from each specimen was connected to 3mm horizontal PVC tube, which subsequently emptied fluid running through the system into a lower collection jar. A submerged motor was placed in the lower jar to pump the fluid into the fluid reservoir placed 20 cm above the specimen to obtain intra pulpal pressure of 20mm of Hg (Figure 1). A pressure gauge was used to monitor the intra pulpal pressure of the specimens. An intra pulpal pressure of 20 mm Hg was maintained before and after bonding procedures while the pressure was reduced to 15 mm of Hg during bonding procedures [ 7 ].

### Bonding procedure

The prepared cavity was etched with 37% phosphoric acid (EazEtch Gel, Anabond Stedman Pharma Research Pvt Ltd, Tamilnadu, India.) for 15 sec and rinsed with water. The forty samples were randomly divided into 4 groups (n=10 for each group). Group 1 [control]:: dentin bonding application without prior therapeutic primer application; group 2: solution-based remineralization solution containing [nHAP] alone; group 3: functional group-based remineralization solution containing [Chi- nHAP]; group 4: carrier-based remineralization solution containing [MS nHAP]. For groups 2,3 and 4, the ultrasonicated experimental solution was agitated on the etched dentin surface for 30-sec and excess wiped off with a cotton pellet. The adhesive resin Single bond 2 (3M ESPE, St. Paul, MN, USA) was then applied to de-ntin and enamel with two adhesive coats and light-cured for 20sec by means of the light-curing unit (3M Espe Elipar). Micro hybrid composite resin (Resto Fill, Anabond Stedman Pharma Research Pvt Ltd; Tamilnadu, India) was then placed incrementally and light-cured for 20 sec with an output intensity of 1200mw/cm^2^.

### Micro tensile test

The basic method of preparing a sample for micro tensile strength was modified because of the presence of class I restoration. After 30 days of remineralization period in intra pulpal pressure simulation model, 40 tooth samples were sectioned mesiodistally into multiple sections, each 1mm in width bucco lingually. The samples were then reduced into tooth slabs containing enamel on both ends containing composite and dentin in the center, and thickness of such sample were reduced to obtain a uniform thickness of 1mm. Acrylic resin cylinders were attached on either side of the sample and tested for failure. The samples were stretched in tension using a universal testing machine (Lloyd LRX: Llyod Instruments, Fareham, Hants, UK) at a cross-head speed of 0.5 mm/min until failure. The tension force at failure was recorded and converted into tensile stress in MPa using computer software. Fractures not extending through the composite and dentin were excluded.

### SEM Specimen Preparation

The remaining two samples from each group were analyzed for morphologic changes within the hybrid layer using scanning electron microscopy (SEM) under the low vacuum mode coupled with an energy-dispersive X-ray spectrometer at 1000x (SEM-EDX; S-570, Hitachi, Tokyo, Japan). The specimens were desiccated by immersing sequentially in 70%, 80%, 90%, and 99% alcohol. Finally, samples were mounted on the aluminum stubs and sputter-coated with gold (JEOL, JFC-1600, Auto fine coater) and observed under SEM at different magnifications.

### FTIR Specimen Preparation

IR spectra were recorded on a Nicolet 5700 FTIR spectrometer, equipped with a Smart Orbit diamond Attenuated Total Reflectance (ATR); the spectral resolution was 4 cm^-1^. The ATR area had a 2 mm diameter. IR radiation penetration was about 2 µm. Data were recorded in the transmittance mode over a 4000 to 500cm^-1^ range at 16cm^-1^ resolution and 32 times scan using an infrared spectrophotometer. Intensity values ratio for the peak in area of 900–1200 cm^-1^ phosphate band and area of amide I band (1590–1720cm^-1^) was taken for calculating mineral to matrix. Intensity ratio of CO_3_ band at 1415 and PO_4_ at 1030 sub band was used to calculate the Carbonate-to-phosphate ratio (C/P). 

### Statistical analysis

The mean value results for each group were presented in a standard graph. A one-way analysis of variance (ANOVA) was used to test significant differences among the micro tensile bond strength values of the different groups.

A Bonferroni test for pairwise comparison was used. Data were analyzed using the SPSS program for Windows (Statistical Package for Social Sciences, release 15 for MS Windows, 2006, SPSS Inc., Chicago, IL, USA).

## Results

### Physicochemical characterization of nanoparticles

**Figure 2:**
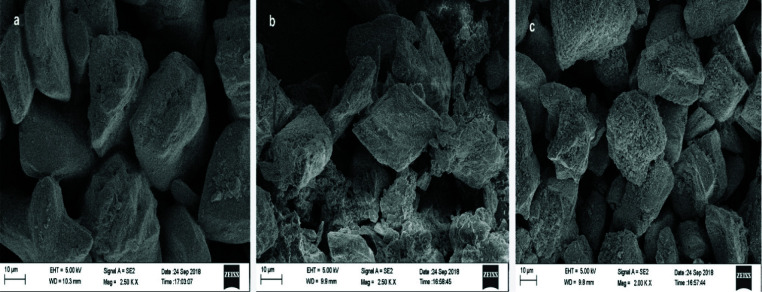
Morphology of different experimental nanoparticle solution observed under SEM at 2500×, **a:** HAP group, **b:** Chi HAP group, **c:** MS -HAP group (hydroxy apatite nanoparticles (Nhap); chitosan + hydroxy apatite nanoparticles (chi-nHAP); mesoporous silica+ hydroxy apatite nanoparticles (MS-nHAP))

**Figure 3:**
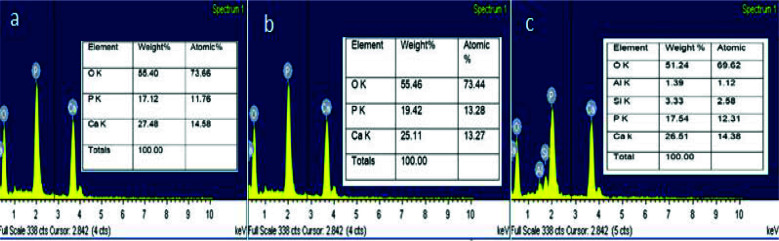
Energy dispersive x-ray (EDX) elemental composition of different experimental nanoparticle solution, **a:** AP group, **b:** Chi HAP group, **c:** MS-HAP group (hydroxy apatite nanoparticles (nHAP); chitosan + hydroxy apatite nanoparticles (chi-nHAP); mesoporous silica+ hydroxy apatite nanoparticles (MS-nHAP))

Nano hydroxyapatite (nHAP): -SEM at 2500× showed approximately hexagonal particle shape and a minimum diameter of 70.42nm (Figure 2a). EDX showed the presence of 27.48 wt% of calcium and 17.12 wt% of phosphorous with a calcium phosphate ratio of 1.60 indicative of nano-hydroxyapatite (Figure 3a). The FTIR spectra of 900-1300 cm^-1^ corresponding to vibrational peaks of phosphate (PO_4_^3-^) were present. The vibration peaks from 1380- 1580 cm^-1^ indicated the presence of the carbonate (CO_3_^2-^) group.

Chitosan with nano-hydroxyapatite (Chi-nHAP): -SEM at 2500× results showed approximately uneven shaped smaller particles on the surface of larger particles. The particles had a minimum diameter of 74.37nm with evidence of agglomeration (Figure 2b-c). The FTIR spectra indicative of chitosan were seen at 1150 to 1,220cmˉ1, indicative of P=O stretching and peak at 1050 to 1100 indicates P–OH group vibrations.

Mesoporous silica with nano-hydroxyapatite (MS-nHAP): -SEM-EDX at 2500× results showed particles with a minimum diameter of 14.65 nm and presence of silica at 3.33wt% and aluminum at 1.39wt% along with nHAP at a calcium phosphate ratio of 1.56 (Figure 3b-c). The FTIR spectra indicative of mesoporous silica were seen at around 1070 cm^-1^ that represents anti-symmetric stretching of the Si-O-Si bonds, and fewer intensity bands at 650 to 800 cm^-1^ also indicate Si-O-Si vibrational mode of silica. FTIR spectra at around 1635 cm^-1^ are associated with the Si–O–H vibration (silanol groups linked to H_2_O molecules).

### Micro tensile bond strength

Table 2 represents the mean micro tensile bond strength of different groups analyzed by one-way ANOVA. The mean micro tensile bond strength values of nHAP Group (22.94±3.457 Mpa), Chi- nHAP Group (23.50± 3.227 Mpa), MS-nHAP Group (24.72±4.189 Mpa) were more than control Group (20.9±2.032 Mpa). Post hoc Bonferroni test revealed significantly higher micro tensile bond strength of experimental groups compared to control. However, there were no significant differences among then nHAP, Chi- nHAP, and MS- nHAP experimental groups.

**Table 2:** Microtensile bond strength of different groups with mean and standard deviation One way ANOVA revealed statistically significant for the groups. Statistically significant if *p*<0.05

Group	N	Mean	Std. Deviation	Std. Error	*p* Value
Control group	10	20.960	2.0326	.6428	0.008^*^
nHAP group	10	22.940	3.4577	1.0934	
Chi-nHAP group	10	23.500	3.2273	1.0206	
MS-nHAP group	10	24.720	4.1899	1.3250	
Total	40	23.030	3.4723	.5490	

Hydroxyl apatite nanoparticles (nHAP); chitosan + hydroxy apatite nanoparticles (chi-nHAP); mesoporous silica+ hydroxy apatite nanoparticles (MS-nHAP)

### SEM analysis of hybrid layer

**Figure 4:**
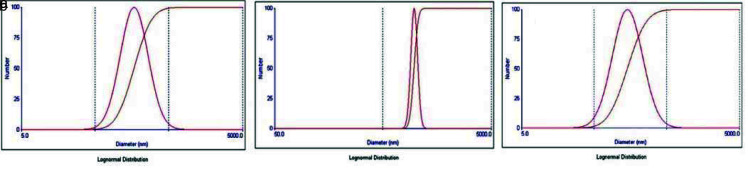
Zeta potential analysis of different experimental nanoparticle solution, **a:** HAP group with mean diameter 185.9 nm, **b:** Chi HAP group mean diameter 323.0 nm, **c:** MS-HAP group mean diameter 183.9 nm (hydroxy apatite nanoparticles (nHAP); chitosan + hydroxy apatite nanoparticles (chi-nHAP); mesoporous silica+ hydroxy apatite nanoparticles (MS-nHAP))

SEM evaluation of hybrid layer was performed at 1000x. MS-nHAP resulted in the uniform deposition of apatite crystal on the surface without any evidence of dentinal tubules openings as compared with control and nHAP group. Chi-nHAP demonstrated uniform deposition of apatite but few opened dentinal tubules were evident (Figure 4).

### FTIR analysis of hybrid layer

**Figure 5:**
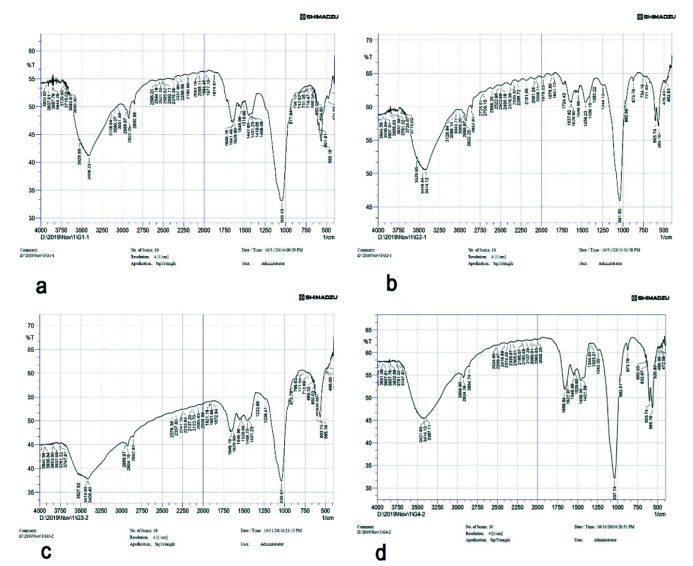
FTIR intensity peaks for ^a:^ control group, ^b:^ nHAP group, ^c:^ ChinHAP, ^d:^ MS-nHAP. Intensity values ratio for the peak in area of 900–1200 cm^-1^ phosphate band and area of amide I band (1590–1720 cm^-1^) was taken for calculating mineral to matrix. Intensity ratio of CO_3_ band at 1415 and PO_4_ at 1030 subband was used to calculate the Carbonate-to-phosphate ratio (C/P) (hydroxy apatite nanoparticles (nHAP); chitosan+hydroxy apatite nanoparticles (chi-nHAP); mesoporous silica+ hydroxy apatite nanoparticles (MS-nHAP))

The intensity peaks of FTIR analysis for different groups are depicted in Figure 5a-d for control, nHAP, Chi-nHAP, and MS-nHAP respectively. The mineral to matrix ratio and carbonate to phosphate ratio of different experimental and control groups are mentioned in Table 3. It can be observed that the MS-nHAP had a higher mineral to matrix ratio compared to other experimental groups, with the control group having the least mineral to matrix ratio. The carbonate to phosphate ratio of 1.46 for the MS-nHAP group indicates the presence of Calcium-deficient hydroxyapatite, Ca/P ratio of 1.3 and 1.28 for Chi-nHAP and nHAP group respectively indicates the presence of octa calcium phosphate.

**Table 3:** Mineral to matrix ratio and carbonate to phosphate ratio of different groups based on the Fourier-transform infrared spectroscopy (FTIR) intensity

Group	Mineral to matrix ratio	Carbonate to phosphate ratio
Control group	11.5	1.69
HAP group	19.8	1.35
Chi-HAP group	32.14	1.30
MS- HAP group	39.52	1.46

Hydroxyl apatite nanoparticles (nHAP); chitosan + hydroxy apatite nanoparticles (chi-nHAP); mesoporous silica+ hydroxy apatite nanoparticles (MS-nHAP)

## Discussion

The micro tensile strength of the hybrid layer obtained with the experimental solutions had significantly better results compared to the control group, where no therapeutic primer/biomimetic remineralization was employed. The micro tensile bond strength among the experimental solution had no significant difference, but overall, the MS-nHAP experimental group had better micro tensile bond strength, followed by the Chi-nHAP group and nHAP group. Thus, the null hypothesis was rejected. 

Since water within the hybrid layer and adhesive resin (termed water treeing) seen in total-etch and self-etch adhesives are responsible for nano leakage and hydrolytic degradation of both resin and collagen, a mechanism has to be developed to replace this water compartment. Recently biomimetic remineralization strategy has been widely used as a process of progressive dehydration mechanism, which attempts to replace the water from the intrafibrillar compartments of collagen fibrils by apatites.

Successful biomimetic remineralization strategy requires a therapeutic primer containing nucleation template and supersaturated calcium phosphate source. To verify this concept and to formulate a new therapeutic primer, we tested the hypothesis of nanoparticle-mediated biomimetic mineralization of acid-etched dentin. Three nanoparticles including nHAP, Chi-nHAP, and MS-nHAP, were employed and tested for micro tensile strength of resin-dentin interface.

Three nanoparticles were employed in this study due to the following advantages and evidence in the literature. MS-nHAP has been extensively used for targeted and sustained release of drugs, proteins, enzymes and genetic materials. Silanol groups on the surface of MS have been used to anchor many functional groups. The advantage of MS-nHAP is related to its pore structure, larger internal surface area, pore-volume, hydrocarbon sorption efficacy, and tunable pore size [ 12 ]. The MS-nHAP has been used as a scaffold because of its biocompatibility, bio-degradability osteoinductive and stimulates type I collagen synthesis [ 13 - 15 ].

Chitosan and its derivative have been used extensively due to its biological activity, excellent biocompatibility, chelating property, antibacterial and complete biodegradability. The chitosan has also been shown to have a collagen cross-linking property, and it has been verified to inhibit matrix metalloproteinases (MMPs) [ 16 ].

Nano-hydroxyapatite has similar morphology, crystal structure, solubility, and biocompatibility compared to dental apatite [ 17 ]. Synthetic nHAP has been shown to remineralize the surface of dentin that has been acid etched and has also been demonstrated to form a new layer of mineralized tissue [ 18 ].

The composition of nanoparticles in three groups: nHAP, Chi-nHAP, and MS-nHAP, were selected after reviewing the literature. The basic composition of these experimental solutions contains 500µg/ml of polyacrylic acid, 200µg/ml of polyvinyl phosphonic acid, and 10 wt% of nHAP dissolved in ethanol. The pH was adjusted to 7.4 with 0.1 M Tris base or 0.1 M HCl [ 2 - 3 , 19 - 21 ]. The dual biomimetic analogs were added to provide a nucleation template and guide the remineralization. The absence of these analogs may not allow remineralization of completely demineralized dentin as in total-etch or more aggressive self-etching systems. Though there is evidence of remineralization of collagen fibrils in partially demineralized dentin, this may be possible due to the presence of remnant seed crystallites, which act as templates for remineralization. For non-classical particle-based crystallization, biomimetic analogs are essential. The addition of dual biomimetic analogs in experimental solution rather than in simulated body fluid is to allow rapid penetration into the dentin and to obtain the function of calcium phosphate stabilizer of polyacrylic acid and as collagen-binding matrix protein of polyvinyl phosphonic acid (PVPA) [ 21 ].

30 wt% of mesoporous silica and 10 wt% of nHAP were employed in this study based on the result of Besinis *et al*. [ 22 ]. The use of the intrapulpal simulation model is to simulate the clinical condition of dentinal fluid perfusion of the resin-dentin interface. The method of immersing the sample completely in the simulated body fluid may result in unexpected remineralization of resin dentin interface, which may be due to excessive/insufficient remineralizing ions reaching the hybrid layer.

Total etch bonding system (Single bond 2) was employed in this study which also contained ethanol as solvent, same as that used in experimental solutions. The total etch bonding system was preferred to know the ability of the experimental solutions to penetrate the demineralized dentin and mineralize the extrafibrillar and intrafibrillar collagen compartments. The reduced hybrid layer durability due to collagen degradation and resin hydrolysis was also shown to be more prevalent with total-etch systems.

Placement of primer containing biomimetic analogs before bonding resin application or use of adhesive resin containing bioactive fillers has demonstrated remineralization of denuded collagen in a short period less than 3 months. Utilizing nanoparticles and biomimetic analogs as a therapeutic primer after acid etching allowed simultaneous penetration into dentinal tubules rather than diffusion from overlying material. This reduced the overall time required for biomimetic remineralization of denuded collagen fibrils. 

**Figure 6:**
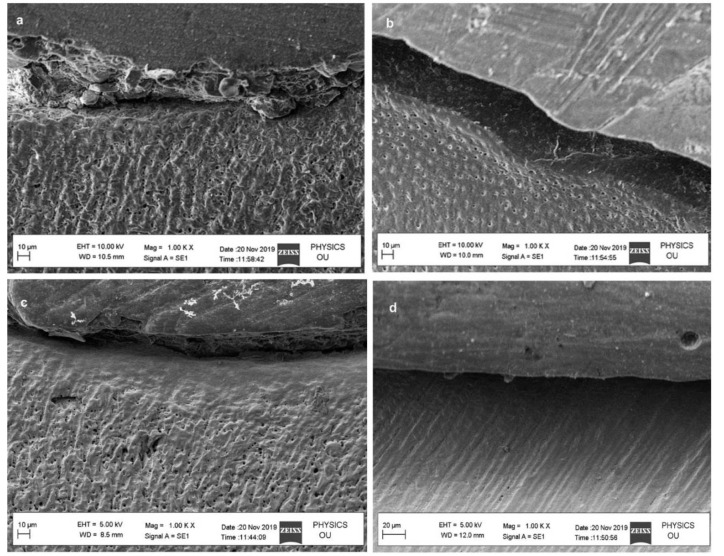
SEM evaluation of hybrid layer observed at 1000×, **a:** Control group with irregular deposition of crystals with many dentinal tubules opening, **b:** nHAP group with fewer dentinal tubules and irregular deposition of apatite, **c:** Chi-nHAP group shows uniform deposition of apatite but with few dentinal tubule openings, **d:** MS -nHAP group shows uniform deposition of apatite crystal on the surface without any evidence of dentinal tubules openings (hydroxy apatite nanoparticles (nHAP); chitosan+ hydroxy apatite nanoparticles (chi-nHAP); mesoporous silica+ hydroxy apatite nanoparticles (MS-nHAP))

The EDX results of all experimental solutions showed a Ca/P ratio near 1.60 in graph (Figure 3), which indicates the stabilization of nHAP in solution by polyacrylic acid. The polydispersity index of all the experimental solutions ranged from 0.005 to 0.27 in Figure 6, which indicates that the nanoparticles have a narrow particle size range and they have low tendency to aggregate.

The FTIR result of all experimental solutions demo-nstrated bands at 3400-3500 cm^-1^ confirming the presence of hydroxyl groups. The bands from 900-1300 cm^-1^ correspond to vibrational peaks of phosphate (PO_4_^3-^). The vibration peaks from 1380–1580 cm^-1^ indicated the presence of carbonate (CO_3_^2-^) group. This indicates that all the groups demonstrated intact hydroxyapatite along with evidence of silica and chitosan. The vibration peaks may shift from higher or smaller wave number positions depending on the preparation route. In addition, the vibrational peaks of chitosan and mesoporous silica may be reduced or absent because of the nH-AP covering the surface of MS-nHAP and Chi-nHAP [ 22 ].

In SEM characterization of MS-nHAP (Figure 6d), one can visualize many smaller particles resulting in a "cauliflower" appearance. This appearance may be due to the presence of nHAP on the surface of Mesoporous silica (MS). The larger particles obtained by the zeta analyzer may be due to the agglomeration of nanoparticles since MS-nHAP and Chi-nHAP were mixtures of two different nanoparticles. It has been shown that the presence of PVPA may reduce the particle size of nanoparticles enabling the nanoparticles to infiltrate dentin [ 1 ].

The nanoparticles were ultrasonicated before application on dentin to overcome the factor of aggregation. The evidence of intrafibrillar mineralization can be obtained with FTIR spectra, which revealed increased mineral to matrix ratio with MS-nHAP followed by Chi-nHAP and nHAP (Table 3). The better result obtained with MS-nHAP was due to the following reasons. The MS has been speculated to have superior dispersion, high solubility, and reactivity, allowing it to penetrate easily into dentinal tubules of 2-3μm diameters. The presence of hydroxyl groups on their surfaces allows them to adhere to the dentin surface [ 12 ]. When MS has been encapsulated with calcium and phosphate source, it results in sustained release of calcium and phosphate ions for long term dentin tubule occlusion and remineralization. Chiang *et al*. [ 23 ] demonstrated calcium phosphate precipitate in the dentinal tubules when MS was loaded with CaO particles and was mixed with 30% phosphoric acid. They showed that in the absence of MS, the supersaturated Ca_2_+/HPO_4_^2−^ got precipitated on the dentinal surface, penetrating to a limited depth of 5-10μm. However, in the presence of MS, that precipitation on the dentinal surface was prevented, an actual precipitation occurred on the surface of MS, and this allowed continued penetration of Ca_2_+/HPO_4_^2−^ ions by the concentration gradient force. The presence of Si peaks in EDX spectra also reflected successful infiltration of a collagen matrix with MS. 

FTIR result of the hybrid layer treated with MS-nHAP, Chi-nHAP, and nHAP revealed the presence of octacalcium phosphate (OCP) in the hybrid layer graph (Figure 5). OCP is often found as an unstable transient intermediate during the precipitation of the thermodynamically more stable calcium orthophosphates in aqueous solutions. The mineral matrix ratio from the FTIR spectra for MS-nHAP also indicated better precipitation of calcium and phosphate deep within the collagen fibrils. The SEM and FTIR results support the increased micro tensile strength values obtained for MS-nHAP. In addition, the FTIR spectra corresponding to amide I, II, and III were more pronounced for control and nHAP compared to Chi-nHAP and MS-nHAP.

The stability of different experimental solutions and compatibility with dentin bonding agent used were not evaluated which account for the limitations of the present study. Further studies should be done evaluating the biocompatibility aspect of the experimental solutions and performance of nano particle based remineralization system in the clinical scenario. 

## Conclusion

Use of Chi-nHAP and MS-nHAP enable sustain release of ca/p precursors for biomimetic remineralization of denuded collagen fibrils. The nHAP particles, which are solution based, showed less infiltration into dentinal tubules. Future attempts at biomimetic remineralization should incorporate carriers such as mesoporous silica and chitosan along with biomimetic analogues for stabilizing the hybrid layer and minimizing its degradation.
